# Clinical and surgical assistance in prostate cancer during the COVID-19 Pandemic: implementation of assistance protocols

**DOI:** 10.1590/S1677-5538.IBJU.2020.S106

**Published:** 2020-07-27

**Authors:** Lara Rodriguez Sanchez, Xavier Cathelineau, Alexis M. Alva Pinto, Ángel Borque-Fernando, Maria Jesús Gil, Chi-Hang Yee, Rafael Sanchez-Salas

**Affiliations:** 1 Université Paris-Descartes L'Institut Mutualiste Montsouris Department of Urology Paris France Department of Urology, L'Institut Mutualiste Montsouris, Université Paris-Descartes, Paris, France; 2 Hospital Universitario Miguel Servet Department of Urology Zaragoza Spain Department of Urology, Hospital Universitario Miguel Servet, Zaragoza, Spain; 3 Clínica Oncosalud Department of Urology Lima Peru Department of Urology, Clínica Oncosalud, Lima, Peru; 4 IIS-Aragon Zaragoza Spain IIS-Aragon, Zaragoza, Spain; 5 The Chinese University of Hong Kong S.H. Ho Urology Centre Department of Surgery Hong Kong Department of Surgery, S.H. Ho Urology Centre, The Chinese University of Hong Kong, Hong Kong

**Keywords:** Prostate cancer, familial [Supplementary Concept], GRADE Approach, COVID-19 [Supplementary Concept], Pandemics

## Abstract

**Purpose:**

Propose an approach of prostate cancer (PCa) patients during COVID-19 pandemic.

**Material and Methods:**

We conducted a review of current literature related to surgical and clinical management of patients during COVID-19 crisis paying special attention to oncological ones and especially those suffering from PCa. Based on these publications and current urological guidelines, a manual to manage PCa patients is suggested.

**Results:**

Patients suffering from cancer are likely to develop serious complications from COVID-19 disease together with an increased risk of postoperative morbidity and mortality. Therefore, the management of oncological patients should be taken into special consideration and most of the treatments postponed.

In case the procedure is not deferrable, it should be adapted to the current situation. While the shortest radiotherapy (RT) regimens should be applied, surgical procedures must undergo the following recommendations proposed by main surgical associations.

PCa prognosis is generally favourable and therefore one can safely delay most of the biopsies up to 6 months without interfering with survival outcomes in the vast majority of cases. In the same way, most of the localised PCa patients are suitable for active surveillance (AS) or hormonal therapy until local definitive treatment could be reconsidered. In metastatic as well as castration resistant PCa stages, adding androgen receptor targeted agents (abiraterone, apalutamide, darolutamide or enzalutamide) to androgen-deprivation therapy (ADT) could be considered in high risk patients. On the contrary, chemotherapy, immunotherapy and Radium-223 must be avoided with regard to the consequence of hematologic toxicity and risk of COVID-19 infection because of immunodepression.

**Conclusions:**

Most of the biopsies should be delayed while AS is advised in those patients with low risk PCa. ADT allows us to defer definitive local treatment in many cases of intermediate and high risk PCa. In regard to metastatic and castration resistant PCa, combination therapies with abiraterone, apalutamide, darolutamide or enzalutamide could be considered. Chemotherapy, Radium-223 and immunotherapy are discouraged.

## INTRODUCTION

### They are called Guidelines, not God's lines

The outbreak of coronavirus that cause the disease COVID-19 has not only created a pandemic situation and a global crisis but beyond imagination, it has completely modified our way to look at medical information and its clinical application.

Medical Guidelines, without a doubt, are of utmost importance and a great deal of work is continuously deployed to offer our patients the highest level of patient care. Every society solidly invests on the training of the young generation to develop novel ideas, then exposing those ideas to a scientific method, eventually obtaining evidence and more importantly reaching a high level of recommendation. Simultaneously, our younger peers are actively taught to verify this information in detail, selecting the best of it and to create a number of standardized practices aiming to objectively guide therapeutic options. Particularly in the case of oncology there are so many exceptions that do not necessarily fit the typical case for one option or another ( 1 ). In these occasions tumor boards and faculty discussions may provide a rational and adoptable treatment option.

Nowadays days we face an unfamiliar enemy, SARS-CoV-2, an RNA virus with low mutational process but a high recombination potential allowing it to switch hosts in a rather timely fashion ( 2 ). Nevertheless, whereas we hold much basic knowledge on the anatomy of this new type of coronavirus which is able to cause severe respiratory illness in 20% of patients (5% of them requiring ventilation and intensive care) ( 3 ), no high level evidence recommendations are available to deal with the challenge it has created to humanity.

Today, we had no choice but to look back at « experience medicine » and use the creator of Sherlock Holmes, Sir Arthur Conan Doyle, approach to identify and solve problems. What do we have in our medical armamentarium that could deal with this threat? Researches around the World are dealing with this question, and of utmost importance, we must understand that during this pandemic, cancer does not stop and some specific patients still need priority treatment.

Our aim is to propose an approach for prostate cancer (PCa) patients management during COVID-19 pandemic.

## MATERIAL AND METHODS

We reviewed most of the ongoing recommendations given by the main health, surgical and urological associations around the World, such as the World Health Organization (WHO), the National Comprehensive Cancer Network (NCCN), the European Association of Urology (EAU) and the British Association of Urological Surgeons ( 3 ߝ 6 ). In addition, publications related to COVID-19 pandemic were also reviewed putting special interest in those focused on surgical management of patients, as well as cancer, in particularly PCa.

Afterwards, the authors propose a practical guide to manage PCa during COVID-19 outbreak based on current urological guidelines. Such proposal is adapted to the special condition we face today.

## RESULTS

### Patient selection to perform a PCa Biopsy and Conditions

It has been reported that SARS-CoV-2 is present in the stool of COVID-19 patients and fecal–oral transmission is possible. While it has not been demonstrated that the prostate biopsy procedure itself would be a way of COVID-19 transmission, we advise to avoid or defer almost all prostate biopsies ( 7 - 9 ).

### Whom to biopsy

In cases where risk factors for high risk PCa are present –prostate specific antigen (PSA) >20, PSA doubling time (PSA-DT) < 6 months, suspicious of clinical T3 disease, and/or local or systemic symptoms-, biopsy can be delayed up to 3 months. On the other hand, in the absence of high risk factors, biopsy may be postponed till 3 to 6 months later ( 6 , 8 ), or even 12 months according to NCCN recommendations ( 7 ) ( Table-1 ).

**Table 1:** Management of clinical suspicion of PCa and localized PCa during the COVID-19 era.

Tumor stage	Recommendations	Comments
**Clinical suspicion (elevated PSA and/or abnormal DRE)**	Presence of High risk PCa factors – PSA >20 – PSA-DT doubling time < 6 months – T3 disease, and/or local or systemic symptoms Biopsy must be delayed up to 3 months ( 8 ). Absence of High risk PCa factors Biopsy may be postponed 3 to 6 months ( 8 ).	PCa prognosis generally favourable can safely delay most biopsies up to 6 to 12 months without interfering with survival outcomes in the vast majority of cases ( 6 - 8 ).
**Localized very low, low and risk favorable intermediate-risk diseases.**	AS must be prioritized while RP as well as RT should be deferred.	NCCN and EAU PCa guidelines currently recommend AS ( 15 , 16 ).
**Localized unfavorable intermediate risk**	Delay local definitive treatment	Delay RP and RT may not imply a very high impact on oncological outcomes ( 13 , 14 ).
	Start ADT 6-monthly formulations	NCCN and EAU PCa guidelines currently recommend short course of ADT added to RT ( 15 , 16 ).
		Preoperative ADT studies show a lack of benefit in prolonging overall survival but an improvement in pathological variables ( 17 ).
**Localized High-risk and very high-risk**	Prioritize definitive treatment if it is available	
	Start ADT 6-monthly formulations	Initiation of ADT must be the standard of care in these patients until local therapy could be reconsidered as the coronavirus crisis improves or ends.
	Consider ADT followed by RT in selected patients	The benefit of neoadjuvant ADT has already been widely verified before RT ( 17 ).
	Consider ADT followed by RP in selected patients	Preoperative ADT studies show a lack of benefit in prolonging overall survival but an improvement in pathological variables ( 17 ).

In regard to Multi-Parametric Magnetic Resonance Imaging (mpMRI), the EAU recommends upfront pre-biopsy mpMRI if resources allow. However, if the patient is suspected to be liable to risk of progression and metastasis, biopsy can be performed without prior MRI ( 6 ).

Considering a possible fecal-oral COVID-19 transmission no rectal lavage for preparation and complete protective personal equipment (PPE) during procedure are advisable. Negative-pressure rooms should be utilized when possible ( 6 - 9 ).

### Patient Approach

In those patients with localized PCa, the two available options to treat them with curative intent, namely radiotherapy (RT) and radical prostatectomy (RP), must be both postponed as much as possible during coronavirus crisis ( 10 , 11 ). In the case of metastatic PCa patients the use of some systemic treatments may be compromised as a consequence of an increase in the number of visits to health centers and the risk of iatrogenic infection that it would entail.

Nowadays the main question to be resolved is how long our patients can wait for a treatment without interfering with oncological outcomes. While this doubt is clarified we propose the following management.

### Localized disease

### Very low, low and risk favorable intermediate-risk diseases

A recent prospective, open-enrollment cohort study showed a risk of cancer death or metastasis lower than 1% over 15yr follow-up in Grade Group 1 PCa patients who underwent Active surveillance (AS) ( 12 ). According to this study as well as PROTECT and PIVOT trials ( 13 , 14 ), NCCN and European PCa guidelines currently recommend or propose AS as a good management option in this group of patients having favorable outcomes( 15 , 16 ). Therefore, AS must be prioritized while RP as well as RT should be deferred until restrictions to contain the spread of COVID-19 are over ( Table-1 ).

Follow-up biopsies and PSA-tests should be postponed by > 3 months from the preplanned appointment in order to decrease the number of visits to hospital and to promote social distancing ( 6 ).

### Unfavorable intermediate risk

Despite the fact that AS for these stages of the disease is not contemplated in the current guidelines ( 15 , 16 ), given the extraordinary situation in which we find ourselves in, RP and RT should be postponed with a believably not very high impact on specific cancer mortality ( 13 , 14 ).

Short course (4-6 months) of androgen--deprivation therapy (ADT) added to RT are indicated in these patients ( 15 , 16 ) while ADT prior to RP might be considered during COVID19 pandemic. Although it is well known that this last approach is not associated with survival benefits it is also related to better pathological results ( 17 ). For these reasons, we recommend the initiation of androgen blockage.

In relation to the possible effect that a prolonged neoadjuvant treatment may have on oncological outcomes it has been observed that extending neoadjuvant ADT therapy duration prior to RT from 8 to 28 weeks neither significantly improve nor worsen oncological outcomes on patients with unfavourable intermediate risk PCa ( 18 ). These results suggest that we may safely delay the need to definitive local treatment for 4-6 months ( 9 , 18 , 19 ) ( Table-1 ).

Keeping into consideration that those patients with a PCa grade group 3 could have an increased risk for eventual metastases ( 19 ) as well as a five-fold increased risk of PCa mortality compared to grade group 2 ( 20 ), we suggest to continue follow-up PSA-tests every 3 months and provide the results by telehealth.

### High-risk and very high-risk

Initiation of ADT must be the standard of care in these patients until local therapy could be reconsidered as the coronavirus crisis improves or ends. The benefit of neoadjuvant ADT has already been widely verified before RT ( 17 ).

In view of a lack of benefit in prolonging overall survival, current urological guidelines strongly discourage the use of elective neo-adjuvant ADT in patients who are going to undergo RP outside of clinical trials ( 15 - 17 ). However, preoperative ADT studies have shown a significant reduction in positive surgical margins and downstaging along with an improvement in other pathological variables such as lymph node involvement. Additionally, these results tended to be better if neo-adjuvant ADT was prolonged from 3 to 6 or 8 months prior to surgery ( 17 ).

Intensive androgen blockage prior to RP is currently under study with favorable preliminary results, but further study is necessary ( 21 ).

In case of detecting patients with a rapid PSA-DT (≤3 months) timely therapy could be indicated and the benefits of immediate treatment must be weighed against the risk associated to iatrogenic exposure to COVID-19 ( 11 ).

As long as there is a limited availability of operating rooms, material and human surgical resources which make it impossible to perform RP, RT could be an alternative. Within RADS (Remote visits, Avoidance, Deferments, and Shortening of radiotherapy) framework created by Radiation Oncologist, shortening of the RT treatment is the fundamental principle for these high risk patients without compromising the oncological outcomes (4, 11 ) ( Table-1 ).

Like what was proposed previously for patients with unfavourable intermediate-risk PCa, we suggest to maintain quarterly PSA monitoring.

### Unfavourable features after radical prostatectomy

According to preliminary results from ARTISTIC meta-analysis presented at ESMO 2019 Congress, event-free survival is not improved with adjuvant radiotherapy (ART) compared to **salvage radiotherapy (SRT)** in patients with combined high-risk features (pT3-T4/R1/GS ( 8 - 10 , 22 ). Despite ART remains the recommended treatment option until more evidence to suggest otherwise ( 15 , 16 ), we strongly advise SRT as a safe alternative ( Table-2 ).

**Table 2:** Management of unfavourable features after radical prostatectomy or biochemical recurrence after local treatment of PCa during the COVID-19 era.

Tumor Stage	Recommendations	Comments
**Unfavourable features after radical prostatectomy**	Avoid adjuvant RT.	According to ARTISTIC meta-analysis, event-free survival is not improved with ART compared to SRT in patients with combined high-risk features (pT3-T4/R1/GS 8-10) ( 22 ).
**Biochemical recurrence**	Delay complementary studies as well as salvage treatments, especially in EAU low-risk cases.	Recent studies suggest that just a subgroups of patients would develop progressive disease following BCR after local treatment ( 23 ).
	Offer salvage treatment for those patients with high-risk BCR if it is available. If not, neoadjuvant ADT could be considered ( 6 ).	

**RT** = Radiotherapy; **ART** = Adjuvant Radiotherapy; **SRT** = Salvage Radiotherapy; **EAU** = European Association of Urology **BCR** = Biochemical Recurrence; **ADT** = Androgen deprivation therapy

### Biochemical recurrence (BCR)

The real impact of BCR in cancer mortality is currently unknown whereas recent studies suggest that just a subgroup of patients would develop progressive disease following BCR after RP with less optimistic results in case of RT failure. In this sense, patients may be stratified into EAU Low-Risk or High-Risk BCR according to PSA-DT, pathological ISUP grade and interval to biochemical failure ( 23 ).

According to the above, in case of clinical suspicion of BCR the authors suggest to postpone complementary studies as well as salvage treatments during COVID-19 pandemic, especially in Low-Risk cases. On the other hand, we propose to offer salvage treatment for those patients with High-Risk BCR if it is available. If not, neoadjuvant ADT could be considered ( 6 ) ( Table-2 ).

### Non metastatic castration- resistant prostate cancer

Three randomised phase III trials, PROSPER, SPARTAN and ARAMIS showed a significant metastatic free survival benefit in non-metastatic castration- resistant PCa patients treated with enzalutamide vs. placebo, apalutamide vs. placebo or darolutamide vs. placebo, respectively. Therefore, current guidelines strongly recommend these drugs to patients with castration- resistant PCa, absence of metastases and PSA-DT < 10 months. Taking into account that survival benefit was not proven after 20 months of follow-up as well as potential adverse events, we recommend these drugs in high selected patients during the COVID-19 pandemic ( 1 , 15 , 16 ) ( Table-3 ).

**Table 3:** Management of non metastatic castration- resistant PCa during the COVID-19 era.

Tumor Stage	Recommendations	Comments
**Nonmetastatic castration- resistant prostate cancer**	Consider combination castration therapy with the new hormonal treatments (apalutamide, darolutamide, enzalutamide) in high selected patients.	These drugs have demonstrated benefits in terms of metastatic free survival in patients with PSA-DT < 10 months ( 1 , 15 , 16 ).

**PSA-DT** = Prostate Specific Antigen - Doubling Time

### Metastatic disease

### Metastatic castration- sensitive prostate cancer

ADT must be initiated according to current standard of care ( 15 , 16 ), with the six-month formulations being the best choice (4, 5 ).

In regard to Intermittent ADT, it requires a closer PSA and testosterone monitoring in addition to possible images so it should be avoided in order to minimize hospital attendance.

In the last few years combination castration therapy with the new hormonal treatments (abiraterone, apalutamide or enzalutamide) has demonstrated benefits in terms of survival compared to ADT alone. Abiraterone acetate and prednisone or apalutamide added to ADT significantly reduce the risk of death by an amount equal to 28 and 33% respectively ( 24 , 25 ) while enzalutamide plus ADT reduces radiographic progression-free survival or deaths by 60% ( 26 ).

The median age of patients who are candidates for combination hormonal treatments is around 70 ( 24 - 26 ). Although age is a potential risk factor for mortality of adult inpatients with COVID-19 ( 27 ) and these new drugs imply a closer follow-up, agreeing with the EAU, we suggest to offer immediate systemic treatment within < 6 moths as long as a correct follow-up by telemedicine can be guaranteed ( 6 ).

In case the use of combined hormonal treatment is contemplated, we suggest to avoid abiraterone since the use of corticosteroids in population infected with SARS-CoV-2 is not yet completely clarified ( 5 , 28 ).

With respect to chemotherapy, it must be avoided as much as possible being replaced by ADT or ADT in combination with androgen receptor targeted agents in order to reduce the number of clinical visits and haematological toxicity without compromising oncological outcomes ( Table-4 ).

**Table 4:** Management of metastatic PCa during the COVID-19 era.

Tumor stage	Recommendations	Comments
**Metastatic castration-sensitive prostate cancer**	ADT 6-months formulations must be initiated.	ADT is the current standard of care ( 15 , 16 ).
	Avoid intermittent ADT.	Intermittent ADT requires a closer PSA and testosterone monitoring in addition to possible images.
	Consider combination castration therapy with the new hormonal treatments (abiraterone, apalutamide or enzalutamide).	These drugs have demonstrated benefits in terms of survival compared to ADT alone ( 24 - 26 ).
	Prefer apalutamide or enzalutamide to abiraterone.	Effect of corticosteroids in population infected with SARS-CoV-2 is not yet clear ( 5 , 28 ).
	Avoid CTx.	CTx is associated with hematological toxicity and implies multiple visits to the hospital ( 6 ).
**Metastatic castration-resistant prostate cancer**	ADT 6-months formulations must be maintained.	ADT maintenance is the current standard of care ( 15 , 16 ).
	Consider combination castration therapy with the new hormonal treatments (abiraterone, enzalutamide).	These drugs have demonstrated benefits in terms of survival compared to ADT alone ( 29 , 30 - 32 ).
	Prefer enzalutamide to abiraterone.	Effect of corticosteroids in population infected with SARS-CoV-2 is not yet clear ( 5 , 27 ).
	Avoid CTx.	CTx is associated with hematological toxicity and implies multiple visits to the hospital ( 6 ).
	Avoid Immunotherapy (Sipuleucel-T).	Sipuleucel-T might cause cytokine release while cytokines as IL-6 have been directly related to the most aggressive forms of COVID-19 (4, 34 ).
	Avoid Radium-223.	Radium-223 is associated with overall survival benefit by 3,6 (in the absence of visceral metastases) compared to ADT alone, but it is also associated to hematologic toxicity ( 35 ).
	Avoid starting denosumab or zoledronic acid.	Denosumab or zoledronic acid have no impact on overall survival but could generate osteonecrosis of the jaw or hypocalcaemia ( 36 , 37 ).
	In those patients under treatment, denosumab may be maintained while zoledronic acid should be delayed.	Denosumab can be administrated in its monthly subcutaneous formulation while zoledronic acid requires monthly hospital intravenous administration.

### Metastatic castration- resistant prostate cancer

For castration-resistant metastatic patients, ADT must be maintained.

Abiraterone significantly improves overall survival among patients who previously receive chemotherapy compared to ADT alone. Nevertheless, improvement in median survival is not more than 5 months ( 29 ). In those patients who have not received chemotherapy, median overall survival is also improved from 30,3 to 34,7 months ( 30 ). Similar results are observed with enzalutamide plus ADT. It has been reported an improvement in median overall survival of 5 months in those patients already treated with chemotherapy( 31 ) and 2 months in chemo-naïve patients ( 32 ).

In case we decide to introduce abiraterone or enzalutamide, we must choose enzalutamide for the reasons previously mentioned ( 5 , 27 ).

Chemotherapy also increases median survival in this type of patient, but we advise against its use during the current crisis (4- 6 , 33 ).

Cytokines as IL-6 have been directly related to the most aggressive form of COVID-19. Hence, Immunotherapy with sipuleucel-T whose more frequently adverse events involve cytokine release, should not be given (4, 34 ).

Radium-223 must be further avoided. Although, it is associated with overall survival benefit by 3,6 months in patients with CRPC without visceral metastases compared to ADT alone, it is also associated to hematologic toxicity (anemia, thrombocytopenia and neutropenia) and monthly risk visits to hospital for intravenous administration ( 35 ).

As a result of the lack of benefit in overall survival with the administration of denosumab or zoledronic acid we propose to delay its introduction due to their potential toxicity (e.g., osteonecrosis of the jaw, hypocalcaemia) ( 36 , 37 ). In those cases where treatment has been already started, denosumab can be maintained in its monthly sub-cutaneous administration while zoledronic acid, which requires monthly hospital intravenous administration, should be delayed ( Table-4 ).

## DISCUSSION

PCa is the second most common cancer in men worldwide (behind lung cancer) and the first one in Europe with a higher incidence in developed countries as a consequence of screening programs ( 38 ). Therefore, this pathology represents an important percentage of the burden of work carried out in Uro-Oncology units, being RP one of the most frequent operating room procedures performed by urologists with a rising trend during recent years ( 39 ).

While the management of PCa was already complex and under constant debate ( 15 , 16 ), the current global pandemic has further complicated the treatment algorithm of this pathology. Additionally, all current recommendations are not based on robust evidence, but mostly expert consensus. In this sense, at most the PCa treatment recommendations in “EAU guidelines recommendations to the COVID 19 era” have level 2-3 evidence ( 6 ).

Elective definitive PCa treatments as RP as well as RT are being cancelled or postponed for an unknown time in view of the following points:

1. Patients suffering from cancer are at increased risk of infection and serious complications from COVID-19 ( 40 ).
2. Unknown SARS-CoV-2 infected patients who are asymptomatic and who have undergone a surgery are more likely to suffer from complications with a mortality rate of 20.5% ( 41 ).
3. Risk of SARS-CoV-2 infection in the surgical team as well as patients (iatrogenic exposure to the virus).
4. Need of hospital resources as PPE, Hospital/ICU beds and ventilators.

In regard to definitive treatment choice, we must keep in mind that replacing most of RP by RT could not be the universal solution. In a well-balanced scenario, both treatments coexist and resources should be adapted to needs. Supposing that all patients suitable to undergo active treatment are treated with RT, treatment waiting time would be dramatically increased resulting in treatment delay. The potential solution may create a new problem. Hence, we advise to considerer RP as a potential curative treatment during and after COVID 19 pandemic. In this sense, it has been shown that in localized low and intermediate-risk PCa patients, 6 to 9 months of delay from biopsy to RP is associated to an increased risk of BCR or clinical recurrence at 5 years lower than 18% and 0.6%, respectively. While in high risk patients the risk of BCR is higher (close to 24% after 9-12 from the biopsy), short term ADT might protect them until surgery in 3-6 months ( 42 ). It is important to take into consideration that the studies which lead us to avoid ADT prior to RP due to a lack of survival benefit (neither a detriment) compared to immediate surgery are the same which support the use of neoadjuvant ADT in those patients whose surgery is forced to be delayed during COVID19 pandemic ( 20 ).

The special situation that urologists face today force all of us not only to think about when but also how we must treat our patients.

All elective visits should be postponed or transitioned to telehealth visits to further reduce exposure risk. For those patients who must be seen in clinic, social distancing should be promoted to ensure minimal contact with staffs and other patients ( 43 ).

Once SARS-CoV-2 was confirmed as global pandemic by WHO, and community transmission was accepted, all patients must be considered suspected cases until proven otherwise. Therefore, we consider that all patients should be tested prior to any surgery. In case it is not possible, telephone interview depicting symptomatic or oligo-symptomatic cases could be an alternative option ( 9 , 44 ). Additionally, in those case where abdominal tomography image is required, thorax imagine should be added at the same time.

Testing of elective patients is recommended within 48 hours prior to surgery. SARS-CoV-2 positive patients or clinically suspected patients should have their CaP intervention postponed as far as possible ( 6 ).

Several studies have proven transmission of different viruses during surgery ( 45 , 46 ). According to a recent publication, this risk could be higher during laparoscopic procedures compared to open ones ( 47 ). This is due to the concentrated aerosol in the abdominal cavity formed during the operation being released suddenly when trocars are removed, small incisions are done or instruments are exchanged ( 48 ). In addition, airborne transmission is possible through intubation and extubation. This fact has led the EAU Robotic Urology Section to propose some recommendations to safeguard the health of the surgical staff ( 9 ).

However, we shouldn't forget that not all urological cancers are PCa. The extent of therapeutic alternatives for PCa in its different stages drive us to considerer all of them during the lack of medical and surgical resources in favour of non-deferrable treatments such as cystectomies, trans-urethral resection of high volume tumours, big mass nephrectomies, or orchiectomies.

## CONCLUSION

As a consequence of COVID-19 pandemic several measures have been taken in order to reduce the fast spread of the virus, to protect health professionals from infection during their work, to guarantee the health of in-patients, and to ensure the availability of health resources to address the vast number of patients suffering from the coronavirus disease. Subsequently, clinical and surgical strategies in Urology have been forced to adapt to the changes brought about by COVID-19.

Since PCa prognosis is generally favorable, we can safely delay most of the biopsies while AS must almost be mandatory in those patients with low risk PCa. Furthermore, the existence of therapeutic alternatives such as ADT allows us to defer definitive local treatment in many cases of intermediate and high risk PCa, assuming a believably not too significant impact on oncological outcomes. In regard to metastatic castration resistant PCa, combination therapies with novel drugs such as abiraterone, apalutamide, darolutamide or enzalutamide should be considered in high risk diseases whereas their secondary effects could be managed by telehealth. Chemotherapy or Radium-223 must be avoided because of haematological toxicity and frequent hospital visits. We advise against the use of Sipuleucel-T given the risk of cytokines reaction.

Nevertheless, each PCa case must be considered individually and the proposed recommendations should constantly adapt to the epidemiological evolution of the situation.

## Abbreviations

PCa: Prostate Cancer
CRPC: Castration Resistant Prostate Cancer
RT: Radiotherapy
RP: Radical Prostatectomy
ADT: Androgen Deprivation Therapy
